# Supplementary material for: Proteogenomics analysis unveils a TFG-RET gene fusion and druggable targets in papillary thyroid carcinomas
Source: Nat Commun. 2020 Apr 28;11:2056. doi: 10.1038/s41467-020-15955-w (PMC7188865; doi:10.1038/s41467-020-15955-w)
Supplement: Supplementary file 1 — Supplementary Information [file 41467_2020_15955_MOESM1_ESM.pdf]

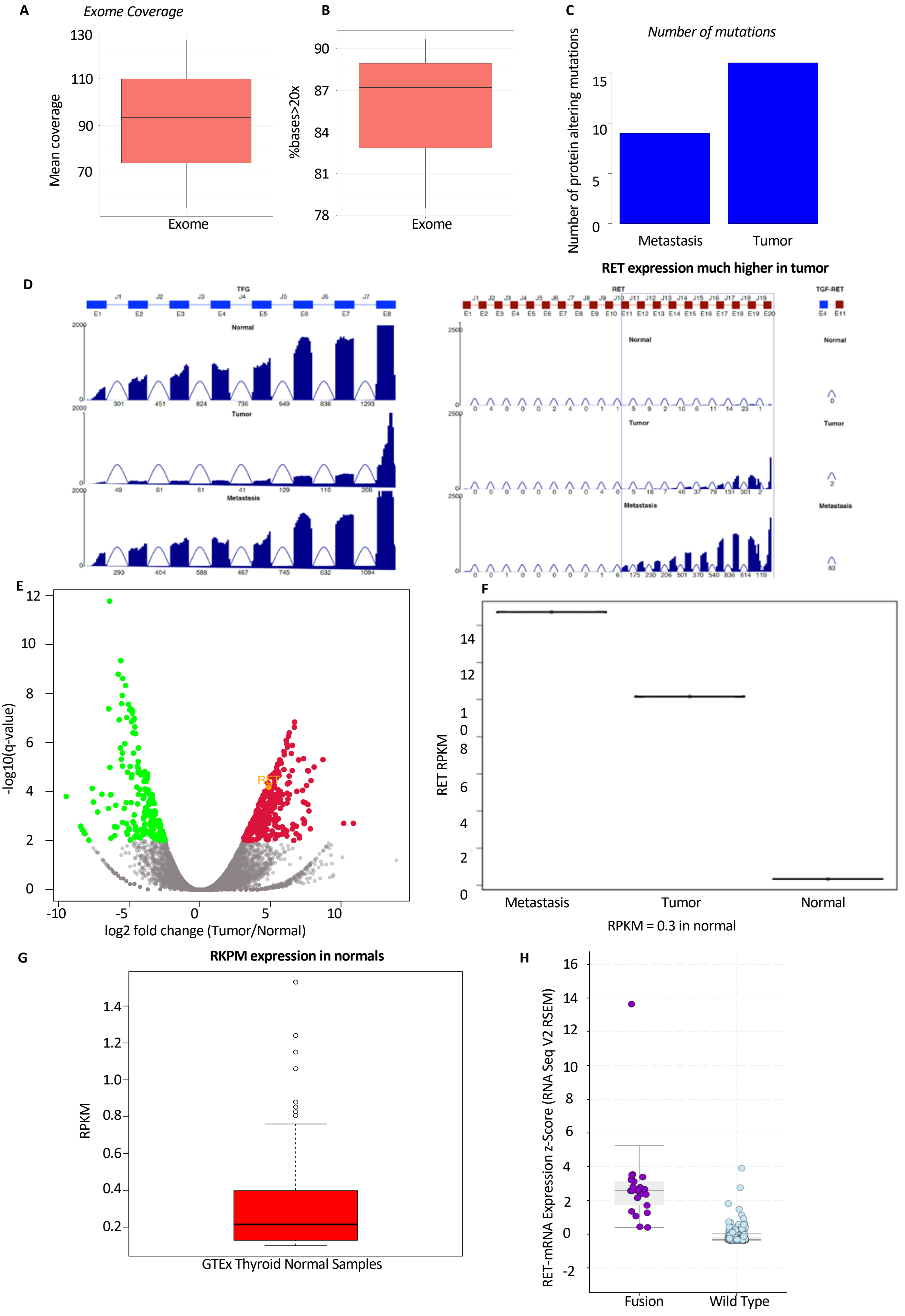

A
Exome Coverage
Mean coverage
130
110
90
70
Exome
B
%bases>20x
90
87
84
81
78
Exome
C
Number of mutations
Number of protein altering mutations
15
10
5
0
Metastasis
Tumor
RET expression much higher in tumor
D
TFG
Normal
Tumor
Metastasis
RET
TGF-RET
E
-log10(q-value)
log2 fold change (Tumor/Normal)
RET
F
RET RPKM
Metastasis
Tumor
Normal
RPKM = 0.3 in normal
G
RKPM expression in normals
RPKM
GTEx Thyroid Normal Samples
H
RET-mRNA Expression z-Score (RNA Seq V2 RSEM)
Fusion
Wild Type

Supplementary Figure 1.

(A) Exome sequencing depth for patient samples (n=3). The median value is shown as a line, with the whiskers extending from the highest value within 1.5 times the interquartile range of the third quartile to the lowest value within 1.5 times the interquartile range of the first quartile.

(B) Exome sequencing percentage of bases covered at minimum of 20x for patient samples (n=3). The median value is shown as a line, with the whiskers extending from the highest value within 1.5 times the interquartile range of the third quartile to the lowest value within 1.5 times the interquartile range of the first quartile.

(C) Number of protein-altering gene mutations detected in exome analysis of LN metastasis and tumor lesions of the patient.

(D) Number of total reads and junction reads found for the normal, tumor and metastasis samples for TFG (left) and RET (right). Right panel is number of junction reads found between exon 4 of TFG exon 11 of RET.

(E) Volcano plot showing significantly down-regulated (green) and up-regulated (red) genes between the tumor and normal sample with the *RET* gene highlighted in orange.

(F) RPMK values for RET expression obtained from RNA-seq analysis of normal (n=1), tumor (n=1) and LN metastasis (n=1) patient tissue. The median value is shown as a line. (G) The average RET expression in normal thyroid tissue from GTEX (http://gtexportal.org) (n=115). The median value is shown as a line, with the whiskers extending from the highest value within 1.5 times the interquartile range of the third quartile to the lowest value within 1.5 times the interquartile range of the first quartile.

(H) Expression of RET in TCGA Papillary Thyroid Carcinomas harboring RET fusions (purple, n=23) or with no RET fusions (light blue, n=365) as given in the cBioPortal (http://cbioportal.org). The median value is shown as a line, with the whiskers extending from the highest value within 1.5 times the interquartile range of the third quartile to the lowest value within 1.5 times the interquartile range of the first quartile.

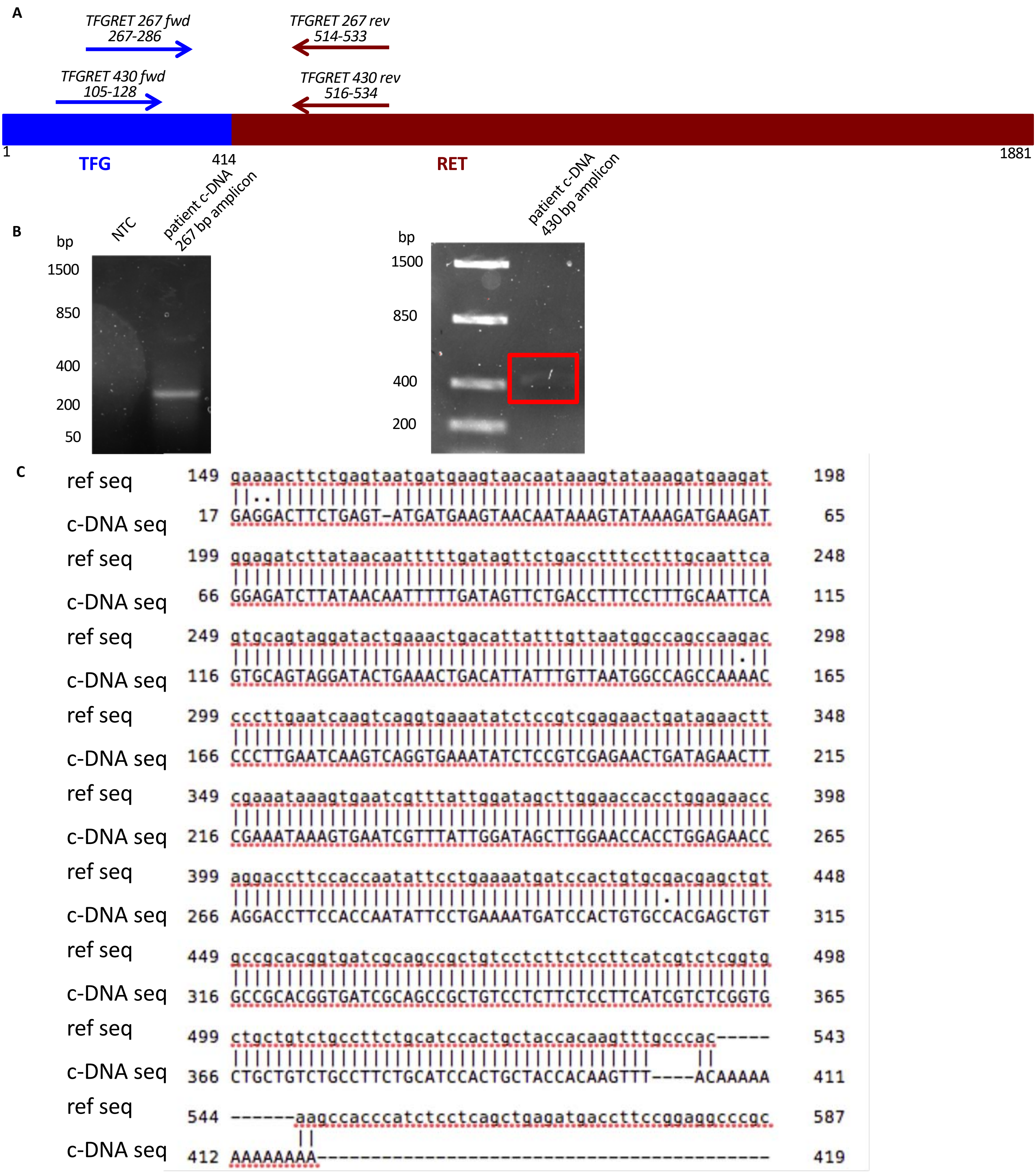

Supplementary Figure 2. Sequencing analysis of patient cDNA.

(A) Schematic of primers used for PCR amplification of a fragment representing the fusion region

(B) cDNA derived from RNA isolated from patient tumor sample was PCR amplified using primers flanking the TFG and RET as described in materials and methods. Both gels electrophoresis reveal the presence of a PCR product around the expected molecular size (from a single representative experiment).

(C) Sequencing analysis of 430bp amplicon confirmed that the PCR product was indeed a TFG-RET transcript.

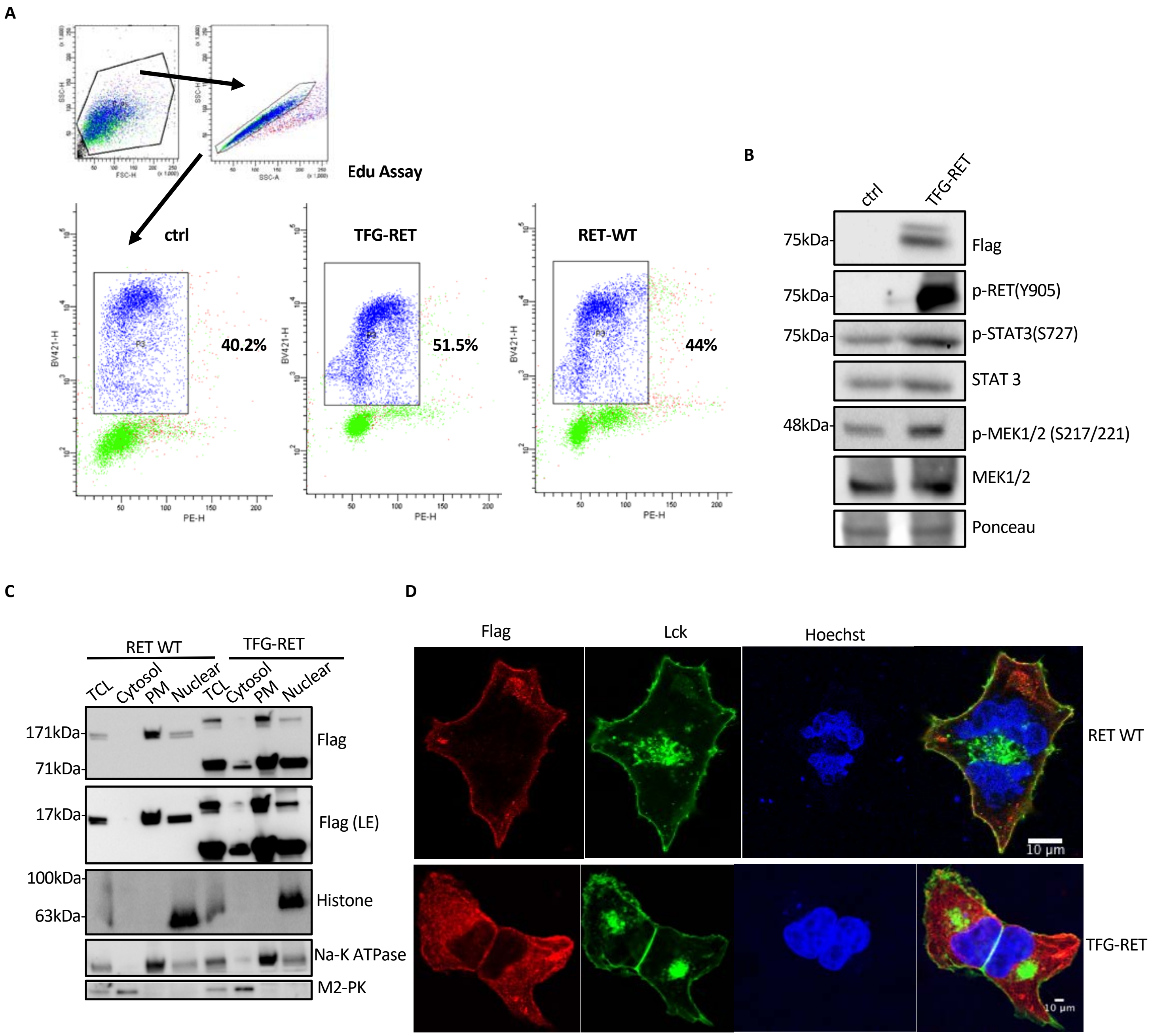

Supplementary Figure 3. TFG-RET localizes in the cytoplasm.

(A) DNA synthesis analysed using Edu assay in Nthy-ori 3-1 cells stably expressing TFG-RET and RET WT. Cells transfected with empty vector served as control. Gating strategy: a SSC-H vs. FSH-H plot was applied to exclude cellular debris. Then a SSC-H vs. SSC-A plot was used to exclude doublets.

(B) Immunoblots for indicated proteins (p, phosphorylated) in lysates from 293T cells transiently expressing FLAG-tagged TFG-RET (pPHAGE C-TAP vector) along with empty vector control (n=3 independent experiments).

(C) Immunoblot analysis of cell lysates from Nthy-ori 3-1 cells stably expressing TFG-RET shows that TFG-RET was also localized in the cytoplasm, while RET wild type (RET WT) was not detected in the cytosolic fraction. Both TFG-RET and RET wild type were detected in the plasma membrane (PM), (tcl, total cell lysate)(LE, longer exposure) (n=3 independent experiments).

(D) Representative images from Nthy-ori 3-1 cells stably expressing TFG-RET (flag tag) or RET WT (FLAG tag) grown on glass coverslips. While TFG-RET (red) exhibits cytosolic distribution, RET (red) expression is not seen in the cytoplasm. GFP-LCK (green) marks the plasma membrane (also, pericentrosome), Hoechst (blue) stains the nucleus (n=3 independent experiments).

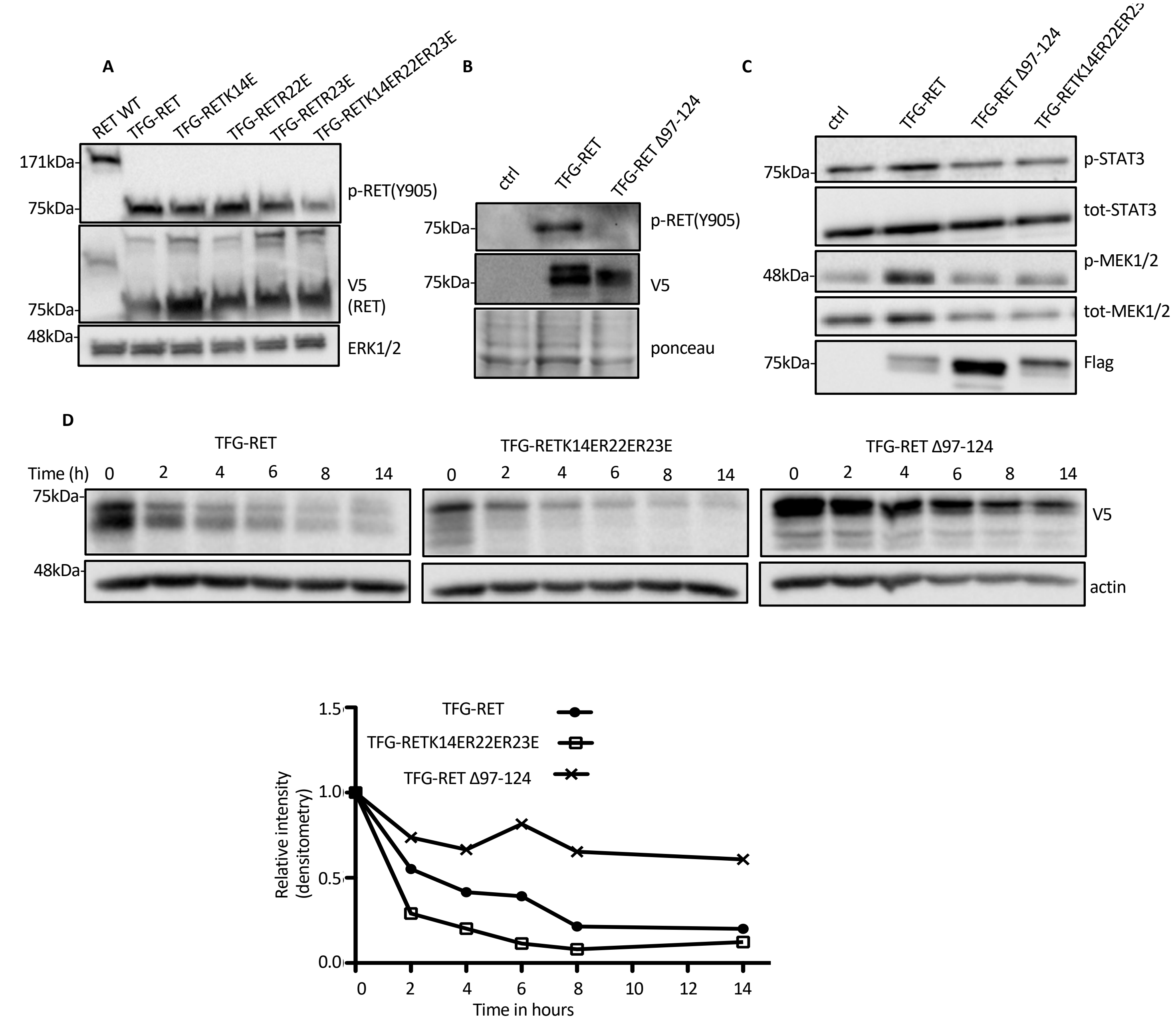

Supplementary Figure 4. TFG domains play a role in RET phosphorylation and stability of TFG-RET.

(A) Mutations in PB1 domain reduces RET autophosphorylation. Western blots for indicated proteins (p, phosphorylated) in lysates from HeLa cells transiently transfected with RET WT, TFG-RET, TFG-RET K14E, TFG-RET R22E, TFG-RET R23E and TFG-RET K14ER22ER23E in pcDNA DEST40 Vector (His and V5-tagged). Shown are data from a single representative experiment.

(B) Western blots for indicated proteins (p, phosphorylated) in lysates from Nthy-ori 3-1 transiently transfected with TFG-RET, TFG-RET Δ97-124 or empty vector control (n=3 independent experiments).

(C) Western blots for indicated proteins (p, phosphorylated) in lysates from Nthy-ori 3-1 transiently transfected with the TFG-RET, TFG-RETK14ER22ER23E, TFG-RET Δ97-124 or with empty vector control (n=3 independent experiments).

(D) Cycloheximide chase assay of Nthy-ori 3-1 cells transiently expressing TFG-RET, TFG-RET K14ER22ER23E, TFG-RET Δ97-124 shows that TFG-RETK14ER22ER23E exhibits lesser protein stability and TFG-RET Δ97-124 exhibits higher protein stability, relative to TFG-RET . Shown are data from a single representative experiment.

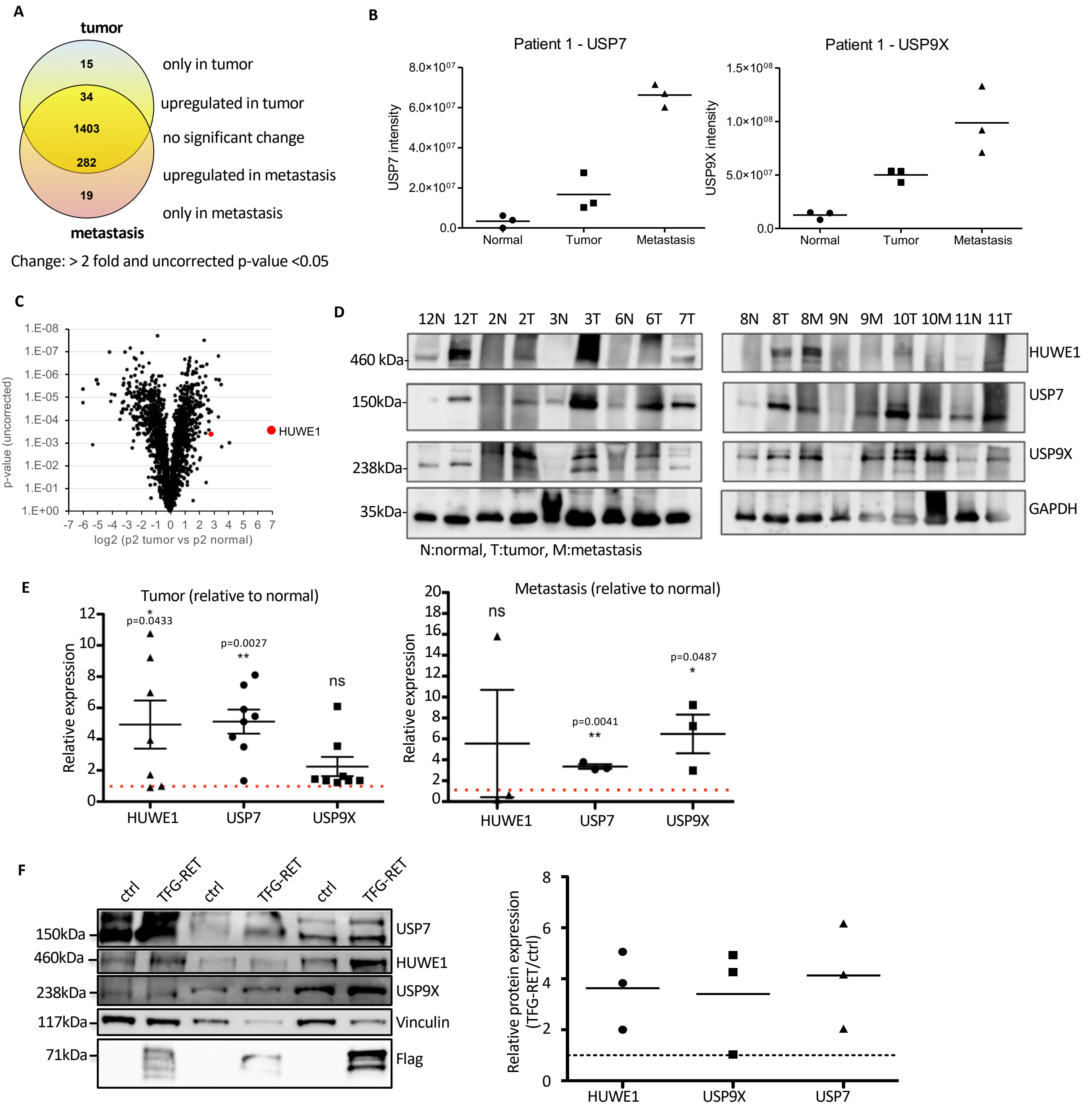
A
tumor
15
34
1403
282
19
metastasis
only in tumor
upregulated in tumor
no significant change
upregulated in metastasis
only in metastasis
Change: > 2 fold and uncorrected p-value <0.05
B
Patient 1 - USP7
USP7 intensity
8.0×10^07
6.0×10^07
4.0×10^07
2.0×10^07
0.0
Normal
Tumor
Metastasis
Patient 1 - USP9X
USP9X intensity
1.5×10^08
1.0×10^08
5.0×10^07
0.0
C
p-value (uncorrected)
1.E-08
1.E-07
1.E-06
1.E-05
1.E-04
1.E-03
1.E-02
1.E-01
1.E+00
-7 -6 -5 -4 -3 -2 -1 0 1 2 3 4 5 6 7
log2 (p2 tumor vs p2 normal)
HUWE1
D
12N 12T 2N 2T 3N 3T 6N 6T 7T
8N 8T 8M 9N 9M 10T 10M 11N 11T
460 kDa
150kDa
238kDa
35kDa
HUWE1
USP7
USP9X
GAPDH
N:normal, T:tumor, M:metastasis
E
Tumor (relative to normal)
Relative expression
p=0.0433
*
p=0.0027
**
ns
HUWE1
USP7
USP9X
Metastasis (relative to normal)
ns
p=0.0041
**
p=0.0487
*
F
ctrl
TFG-RET
150kDa
460kDa
238kDa
117kDa
71kDa
USP7
HUWE1
USP9X
Vinculin
Flag
Relative protein expression
(TFG-RET/ctrl)
HUWE1
USP9X
USP7

Supplementary Figure 5. Ubiquitin pathway associated proteins are up-regulated in patients PTC and TFG-RET expressing cells

(A) Venn diagrams representing the differential expression of proteins as seen in the mass spectrometric data from the TFGRET patient's normal, tumor and metastatic lesions (n=1). The absolute number of proteins identified in each sample (normal vs tumour vs metastasis) is depicted.

(B) Intensity values of indicated proteins detected in each of the three mass spectrometry runs of protein samples from normal, tumor and metastasis lesions of patient 1.

(C) Volcano plot showing differentially expressed proteins as seen in the mass spectrometric data from patient #2 (n=1).

(D) Western blot analysis of HUWE1, USP7 and USP9X from protein lysates extracted from fresh frozen patient tissue (n=9). Samples were labelled as N – Normal, T – Tumor, M- Metastasis.

(E) Expression of HUWE1, USP7 and USP9X was quantified from (C): first ratio protein of interest/GAPDH was calculated . Tumor and metastasis samples were then normalized to control samples. Both HUWE1 and USP7 showed a significant upregulation in tumor compared to normal tissue (error bars=SEM, n=8 for USP7 and USP9X, n=7 for HUWE1, paired t test, two-tailed, ** p value < 0.01, * p value < 0.05, ns not significant). USP7 and USP9X display a significant increase in their expression in metastatic samples compared to control (error bars=SEM, n=3, paired t test, two-tailed, ** p value < 0.01, * p value < 0.05, ns not significant). The expression correlates to the intensity of the bands from the western blots as described in the methods.

(F) Cell lysates of N-Thy-Ori 3-1 stably transfected with FLAG-tag expressing pPHAGE C-TAP TFG-RET along with empty vector control and were subjected to immunoblot analysis. HUWE1 expression was quantified from immunoblot. Error bars represent ± SEM (n=3).

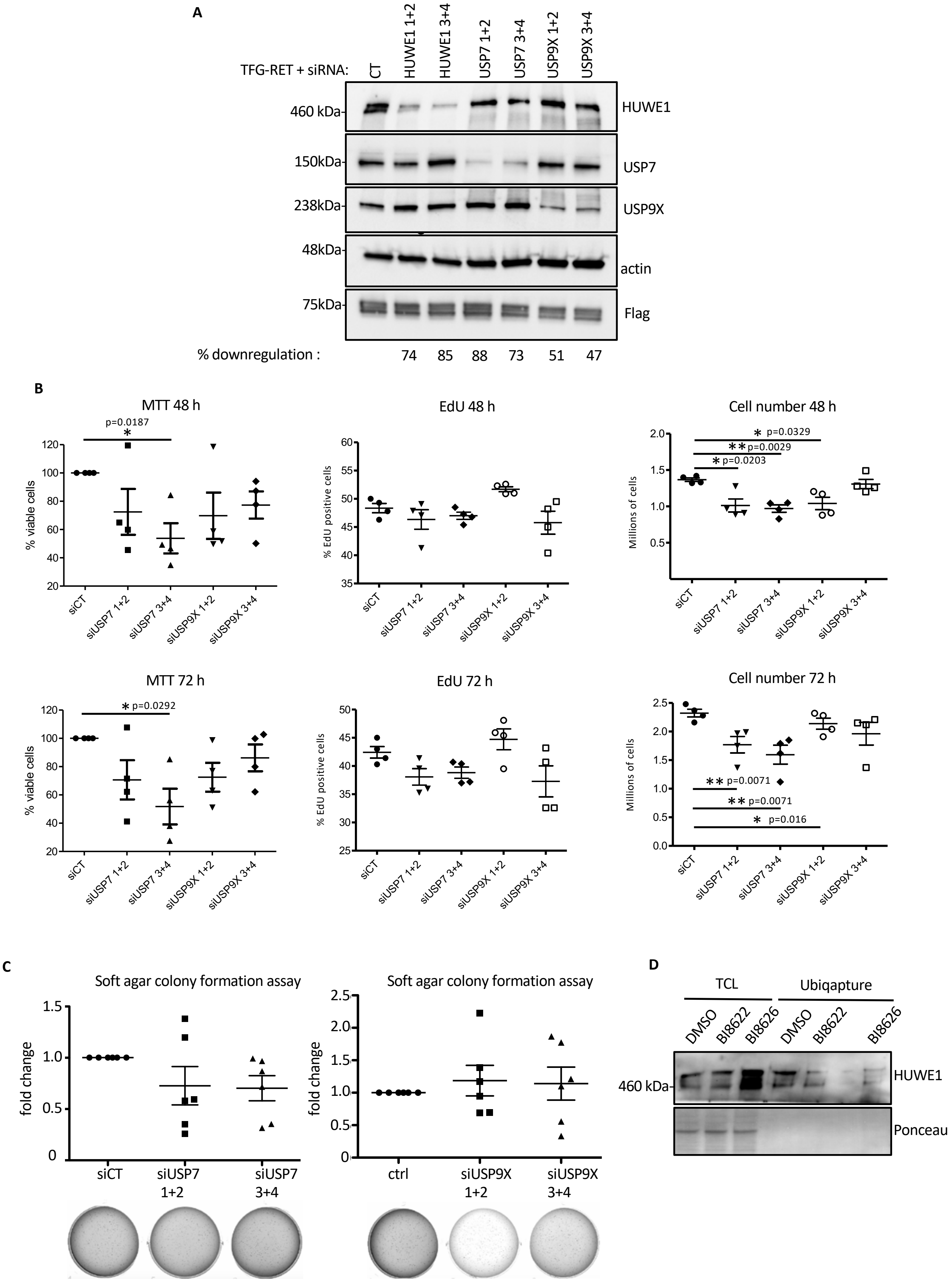

A
TFG-RET + siRNA:
CT
HUWE1 1+2
HUWE1 3+4
USP7 1+2
USP7 3+4
USP9X 1+2
USP9X 3+4
460 kDa
HUWE1
150kDa
USP7
238kDa
USP9X
48kDa
actin
75kDa
Flag
% downregulation :
74 85 88 73 51 47
B
MTT 48 h
p=0.0187
% viable cells
EdU 48 h
% EdU positive cells
Cell number 48 h
p=0.0329
p=0.0029
p=0.0203
Millions of cells
siCT
siUSP7 1+2
siUSP7 3+4
siUSP9X 1+2
siUSP9X 3+4
MTT 72 h
p=0.0292
EdU 72 h
Cell number 72 h
p=0.0071
p=0.0071
p=0.016
C
Soft agar colony formation assay
fold change
siCT
siUSP7 1+2
siUSP7 3+4
ctrl
siUSP9X 1+2
siUSP9X 3+4
D
TCL
Ubiqapture
DMSO
BI8622
BI8626
460 kDa
HUWE1
Ponceau

Supplementary Figure 6. Transient knockdown of USP7 and USP9X leads to reduced cell proliferation and transformation

(A) Western blot of indicated proteins in lysates from Nthy-TFG-RET cells showing reduced protein expression upon siRNA mediated knockdown. Downregulation was checked for each set of experiments. Shown are representative western blots from a single experiment.

(B) MTT assay and cell proliferation in Nthy-TFG-RET transiently transfected with siRNAs against USP7 and USP9X and analyzed as described in figure 5. (error bars=SEM, n=4, paired t-test, two tailed distribution, p values: * <0.05, ** <0.01, ***< 0.0001, ns-not significant). For MTT assay, shown here are the percentage of viable cells and in the Edu assays shown are the percentage of cells in S phase. In the cell counting experiments, the absolute viable cell numbers were shown.

(C) USP7 and USP9X were transiently depleted using siRNAs in Nthy-ori 3-1 cells stably expressing TFG-RET and were cultured in soft agar for 2 weeks followed by staining with crystal violet. USP7 knockdown also showed a reduction in number of colonies transformed, although this effect was not significant. Knockdown of USP9X did not lead to a significant reduced colony formation. Error bars represent ± SEM (n=3 independent experiments with 2 technical replicates, paired t-test, two-tailed).

(D) Western blot analysis of cell lysates of Nthy-TFG-RET cells treated with HUWE1 inhibitors BI8622 and BI8628 (2 h) followed by MG132 treatment (5 h). Ubiquitinylated proteins were isolated using UBI-QAPTURE-Q kit followed by detection of HUWE1. Treatment with BI8622 and BI-8626 reduced the ubiquitination of HUWE1. Shown are representative western blots from a single experiment which was repeated with identical results once more.

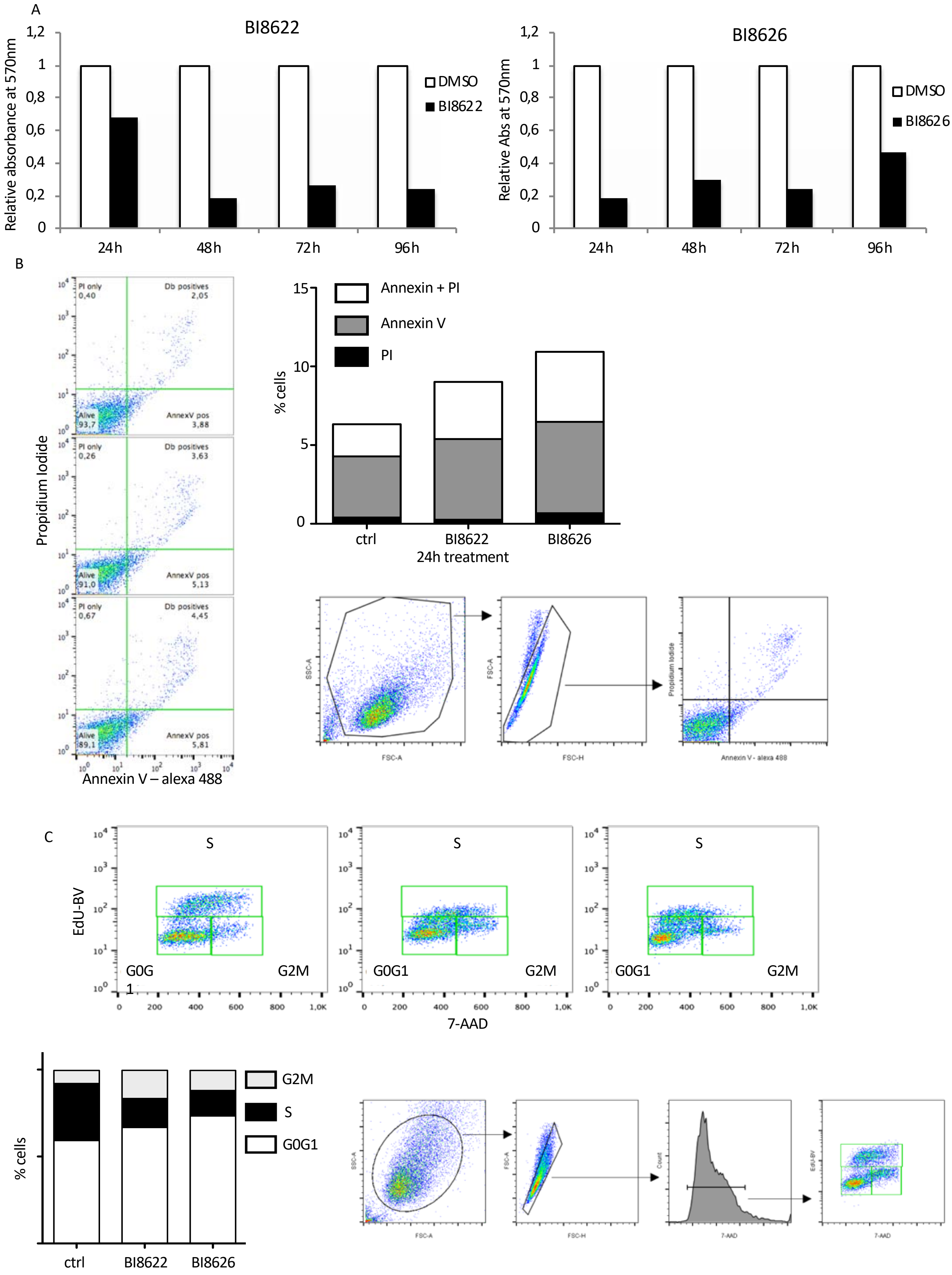

A
BI8622
Relative absorbance at 570nm
DMSO
BI8622
24h
48h
72h
96h
BI8626
Relative Abs at 570nm
DMSO
BI8626
B
Propidium Iodide
Annexin V – alexa 488
PI only
Db positives
Alive
AnnexV pos
0,40
2,05
93,7
3,88
0,26
3,63
91,0
5,13
0,67
4,45
89,1
5,81
Annexin + PI
Annexin V
PI
% cells
ctrl
BI8622
BI8626
24h treatment
SSC-A
FSC-A
FSC-H
Propidium Iodide
Annexin V - alexa 488
C
EdU-BV
S
G0G
1
G0G1
G2M
7-AAD
% cells
G2M
S
G0G1
ctrl
BI8622
BI8626
Count

Supplementary Figure 7. HUWE1 inhibitors inhibit cell proliferation of TFG-RET cells, but do not induce cell death

(A) Nthy-Ori 3-1 cells stably transfected with FLAG tag expressing pPHAGE C-TAP TFG-RET were seeded in 96 well plates and treated with 20 μM BI8622 or BI8626, along with DMSO as control. Cellular viability/proliferation was assessed in an MTT assay at indicated time points. Both BI8622 and BI8626 reduced cell proliferation at all the time points checked.

(B) Nthy-TFG-RET cells were treated with 20 μM BI8622 and 20 μM BI8626 along with DMSO as control for 24 h. Cells were harvested after treatment, following propidium iodide and annexin staining, cells were subjected to flow cytometric analysis. Both BI8622 and BI8626 did not induce strong cell death. Gating strategy: a SSC-A vs. FSH-A plot was applied to exclude cellular debris. Then a FSC-H vs. FSC-A plot was used to exclude doublets.

(C) The effect of BI8622 and BI8626 on cell cycle progression was analysed by cell cycle analysis using EdU (5-ethynyl-2′-deoxyuridine). Nthy-TFG-RET cells were treated with 20 μM BI8622 and 20 μM BI8626 for 24 h. Gating strategy: first a SSC-A vs. FSH-A plot was applied to exclude cellular debris. Then a FSC-H vs. FSC-A plot was used to exclude doublets. Finally 7-AAD (7-Aminoactinomycin D) was used for excluding non-viable cells.
