# Supplementary material for: Proteogenomics analysis unveils a TFG-RET gene fusion and druggable targets in papillary thyroid carcinomas
Source: Nat Commun. 2020 Apr 28;11:2056. doi: 10.1038/s41467-020-15955-w (PMC7188865; doi:10.1038/s41467-020-15955-w)
Supplement: Supplementary file 3 — Description of Additional Supplementary Files [file 41467_2020_15955_MOESM3_ESM.docx]

**Description of Additional Supplementary Files**

**Supplementary Data 1**: The number of total reads for TFG, RET and the fusion junction from normal, tumour and metastasis samples.

**Supplementary Data 2**: RNA sequencing analysis of patient #1 . Shown are data from normal , tumour and metastasis samples

**Supplementary Data 3**: The list of differentially expressed genes (224 in total between Normal and tumour samples)

**Supplemetary Data 4**: Label free quantitative proteomic analysis of normal vs tumour vs metastasis of Patient #1

**Supplementary Data 5**: Proteogenomic analysis of patient #1. The quantitative proteomics data and the RNA seq data were integrated to identify factors that were differentially regulated both at their protein and mRNA levels.

**Supplementary Data 6**: Quantitative proteomic analysis of patient #2-#4.
