# Supplementary material for: Proteogenomics analysis unveils a TFG-RET gene fusion and druggable targets in papillary thyroid carcinomas
Source: Nat Commun. 2020 Apr 28;11:2056. doi: 10.1038/s41467-020-15955-w (PMC7188865; doi:10.1038/s41467-020-15955-w)
Supplement: Supplementary file 10 — Reporting Summary [file 41467_2020_15955_MOESM10_ESM.pdf]

nature research

Corresponding author(s): Krishnaraj Rajalingam

Last updated by author(s): YYYY-MM-DD

Reporting Summary

Nature Research wishes to improve the reproducibility of the work that we publish. This form provides structure for consistency and transparency in reporting. For further information on Nature Research policies, see Authors & Referees and the Editorial Policy Checklist .

Statistics

For all statistical analyses, confirm that the following items are present in the figure legend, table legend, main text, or Methods section.

n/a	Confirmed	
☐	☒	The exact sample size (*n*) for each experimental group/condition, given as a discrete number and unit of measurement
☒	☐	A statement on whether measurements were taken from distinct samples or whether the same sample was measured repeatedly
☐	☒	The statistical test(s) used AND whether they are one- or two-sided<br>*Only common tests should be described solely by name; describe more complex techniques in the Methods section.*
☒	☐	A description of all covariates tested
☒	☐	A description of any assumptions or corrections, such as tests of normality and adjustment for multiple comparisons
☐	☒	A full description of the statistical parameters including central tendency (e.g. means) or other basic estimates (e.g. regression coefficient) AND variation (e.g. standard deviation) or associated estimates of uncertainty (e.g. confidence intervals)
☐	☒	For null hypothesis testing, the test statistic (e.g. *F*, *t*, *r*) with confidence intervals, effect sizes, degrees of freedom and *P* value noted<br>*Give P values as exact values whenever suitable.*
☒	☐	For Bayesian analysis, information on the choice of priors and Markov chain Monte Carlo settings
☒	☐	For hierarchical and complex designs, identification of the appropriate level for tests and full reporting of outcomes
☐	☒	Estimates of effect sizes (e.g. Cohen's *d*, Pearson's *r*), indicating how they were calculated

Our web collection on statistics for biologists contains articles on many of the points above.

Software and code

Policy information about availability of computer code

Data collection	ChemiDoc™ Touch Gel Imaging System, Biorad; Image lab software
Data analysis	Burrows-Wheeler Alignment tool (BWA) (http://maq.sourceforge.net.),<br>Strelka (ftp://strelka@ftp.illumina.com/)<br>Genomic Short-read Nucleotide Alignment Program (http://share.gene.com/gmap)<br>PEAKS v8.5 (Bioinfomatics Solutions Inc, Toronto, CA)<br>(ImageJ (Version: 2.0.0-rc-65/1.51u)<br>Image Lab Version 5.2.1 build 11 (© 2014 Bio-Rad Laboratories)<br>Microsoft®Excel® for Mac 2011, Version 14.7.3 (170325)<br>GraphPad Prism (Prism 5 for Mac OS X, Version 5.0a)

For manuscripts utilizing custom algorithms or software that are central to the research but not yet described in published literature, software must be made available to editors/reviewers. We strongly encourage code deposition in a community repository (e.g. GitHub). See the Nature Research guidelines for submitting code & software for further information.

Data

Policy information about availability of data

All manuscripts must include a data availability statement. This statement should provide the following information, where applicable:

- Accession codes, unique identifiers, or web links for publicly available datasets
- A list of figures that have associated raw data
- A description of any restrictions on data availability

All raw data are available upon request: the proteomics data are deposited in PRIDE (Project accession: PXD016739 and PXD016828). Sequencing data was submitted to the EBI ENA database under accession id PRJEB37220 (ERP120526).

Field-specific reporting

Please select the one below that is the best fit for your research. If you are not sure, read the appropriate sections before making your selection.

- [x] Life sciences
- [ ] Behavioural & social sciences
- [ ] Ecological, evolutionary & environmental sciences

For a reference copy of the document with all sections, see nature.com/documents/nr-reporting-summary-flat.pdf

Life sciences study design

All studies must disclose on these points even when the disclosure is negative.

Sample size	The sample size is always given in the figure legends. We performed technical and biological replicates as indicated in the figure legends. No power calculations based on expected effect sizes were done.
Data exclusions	not done
Replication	The experiments were performed multiple times and it is indicated in the legends now. When we present representative data from a single experiment, which was reproduced, it is indicated (for instance with Western blot experiments).
Randomization	Samples/organisms/participants were not randomized. Established cell lines were used for in vitro experiments. In the animal experiments animals received either control or knock-out cells. Tumor bearing animals were not treated with any drug, so a randomization of the animals was not necessary. Patient material was obtained from the Biobank at the University Medicine Center. We received tissue from papillary thyroid carcinoma patients who did not harbor BRAF, KRAS, NRAS or HRAS mutations. BRAF as well as NRAS mutations are well known driver mutations and we wanted to identify novel mutations contributing to the tumorigenesis of PTCs.
Blinding	not applicable for our study

Reporting for specific materials, systems and methods

We require information from authors about some types of materials, experimental systems and methods used in many studies. Here, indicate whether each material, system or method listed is relevant to your study. If you are not sure if a list item applies to your research, read the appropriate section before selecting a response.

Materials & experimental systems

n/a	Involved in the study
☐	☒ Antibodies
☐	☒ Eukaryotic cell lines
☒	☐ Palaeontology
☐	☒ Animals and other organisms
☐	☒ Human research participants
☒	☐ Clinical data

Methods

n/a	Involved in the study
☒	☐ ChIP-seq
☐	☒ Flow cytometry
☒	☐ MRI-based neuroimaging

Antibodies

Antibodies used

anti-thyroglobulin (M0781, Dako)
anti-phospho-Tyrosine (P-Tyr-1000) MultiMab™ (8954S,CST)
anti-Myelin Basic Protein (MBP) (13344,CST)
anti-V5 antibody (Invitrogen R960-25)
anti-FLAG® M2-Peroxidase (A8592, Sigma)
anti-phospho-RET (Y905) (3221S,CST)
anti-RET (C31B4)(3223S, CST)
anti-phospho-STAT3 (Ser727)(9134P,CST)
anti-STAT3 (9139P, CST)
anti-phospho-Akt (Thr308) (9275, CST)
anti-Akt (C67E7) (4691, CST)
anti-phospho MEK1/2 (9154, CST)
anti-MEK1 antibody (2352,CST)
anti-phospho-p44/42 MAPK (Thr202/Tyr204) (ERK1/2) (9101L, CST)
anti- p44/42 MAPKinase (ERK1/2) (9102, CST)
anti-M2-PK (S-1, Schebo Biotech)
anti-histone H3 (4499S, CST)

	anti-actin (ab49900, abcam)<br>anti-vinculin (SAB4200080, Sigma)<br>anti-GAPDH (G8795, Sigma)<br>anti-HUWE1 (A300-486A, Bethyl)<br>anti-USP7 (A300-033A, Bethyl)<br>anti-USP9X (A301-351A, Bethyl)<br>anti-KRAS (sc-166691, Santa Cruz)<br>anti-Na-K ATPase (MA3-928, Thermo Fisher Scientific)
Validation	In our study we used primarily monoclonal antibodies which were validated for human proteins and for the experimental setup we chose. For fusion proteins, we used a tag-specific antibody as well as a protein-specific antibody. In addition, we conducted several knock-down studies, wherein the specificity of the antibody was shown.

Eukaryotic cell lines

Policy information about cell lines

Cell line source(s)	Nthy-ori 3-1 cells (90011609, Sigma), HeLa (DSMZ) and 293T cells (a kind gift from Dr. Andreas Ernst)
Authentication	Nthy-ori 3-1 cells were provided by a commercial vendor. HeLa cells were authenticated by DSM by multiplex PCR and cytogenetic methods. 293T cells were not authenticated.
Mycoplasma contamination	Mycoplasma was tested routinely in the lab and the contaminant cell lines are either cured are culled from the analysis.
Commonly misidentified lines<br>(See ICLAC register)	*Name any commonly misidentified cell lines used in the study and provide a rationale for their use.*

Animals and other organisms

Policy information about studies involving animals; ARRIVE guidelines recommended for reporting animal research

Laboratory animals	NOD.CB17-Prkdcscid mice, female, age at the beginning of the experiment 8 to 10 weeks
Wild animals	none
Field-collected samples	none
Ethics oversight	The animal experiments were granted by the Landesuntersuchungsbehörde Koblenz - the locally responsible authority for conducting animal studies.

Note that full information on the approval of the study protocol must also be provided in the manuscript.

Human research participants

Policy information about studies involving human research participants

Population characteristics	We obtained material from the local biobank. We used patient material from duly consented PTC patients without known BRAF, KRAS, NRAS or HRAS mutations.
Recruitment	The tissues employed in this study were obtained from the biobank. as a donation with due consent following the ethical and legal guidelines of the institution.
Ethics oversight	University medical Center Mainz, Ethik Komission; ethical approval was obtained from the Landesärztekammer Rhineland Palatinate (Study number: 837.119.15 (9888)).

Note that full information on the approval of the study protocol must also be provided in the manuscript.

Flow Cytometry

Plots

Confirm that:

- [x] The axis labels state the marker and fluorochrome used (e.g. CD4-FITC).
- [x] The axis scales are clearly visible. Include numbers along axes only for bottom left plot of group (a 'group' is an analysis of identical markers).
- [ ] All plots are contour plots with outliers or pseudocolor plots.
- [x] A numerical value for number of cells or percentage (with statistics) is provided.

Methodology

Sample preparation	For the Edu assay established cell lines were used. For the staining itself we employed Click-iT® Plus EdU Pacific Blue™ Flow Cytometry Assay Kit (Thermo Fisher Scientific, C10636).
Instrument	BD FACSCanto, LSRII and Symphony were used for data acquisition.
Software	Data acquisition was done by FACSDiva software. Data were evaluated either by acquisition software or by FlowJo software.
Cell population abundance	For FACS analysis, Nthy-ori 3 cell lines stably expressing either empty vector control or RET-TFG fusion constructs were used. The cell lines were derived by selection with puromycin.
Gating strategy	Gating strategy: a SSC-H vs. FSH-H plot was applied to exclude cellular debris. Then a SSC-H vs. SSC-A plot was used to exclude doublets.

☒ Tick this box to confirm that a figure exemplifying the gating strategy is provided in the Supplementary Information.
