# Supplementary material for: Proteogenomics analysis unveils a TFG-RET gene fusion and druggable targets in papillary thyroid carcinomas
Source: Nat Commun. 2020 Apr 28;11:2056. doi: 10.1038/s41467-020-15955-w (PMC7188865; doi:10.1038/s41467-020-15955-w)

Figure 2A

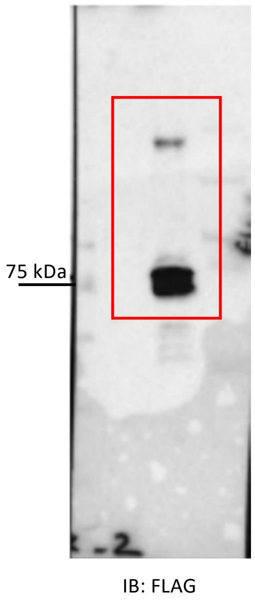

Figure 2B

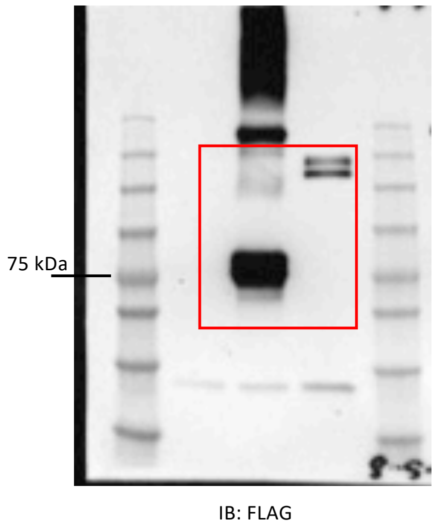

Figure 2D

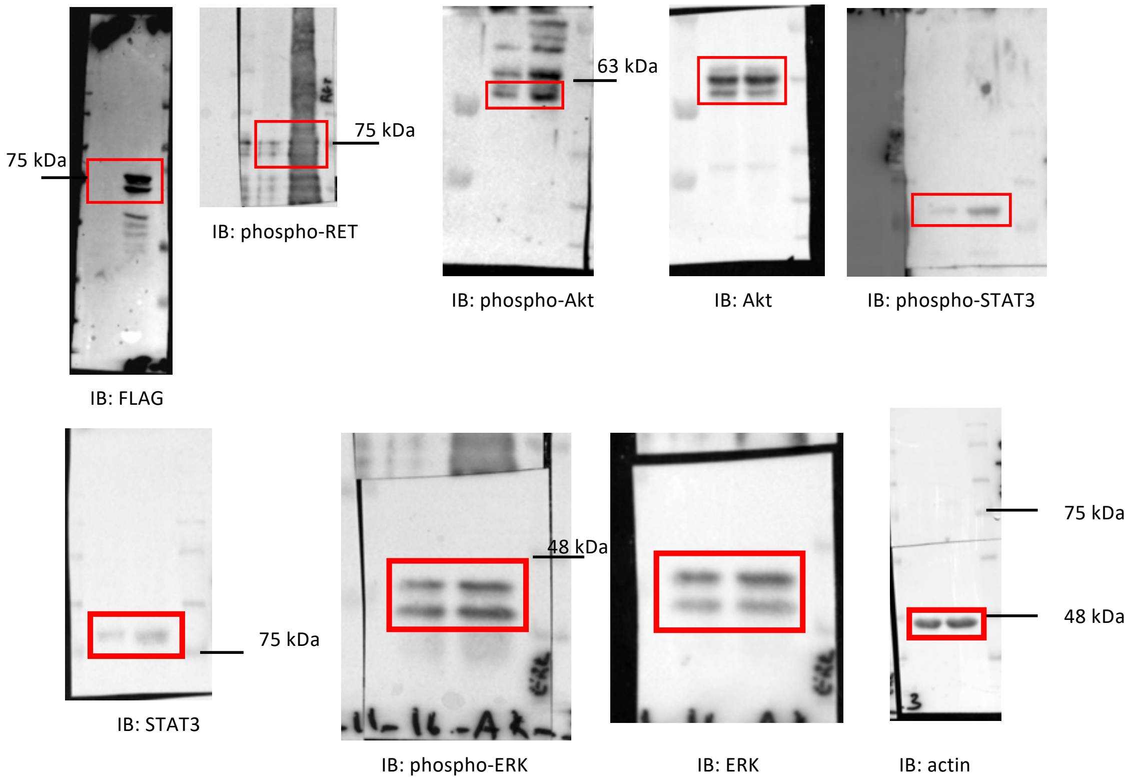

Figure 3A

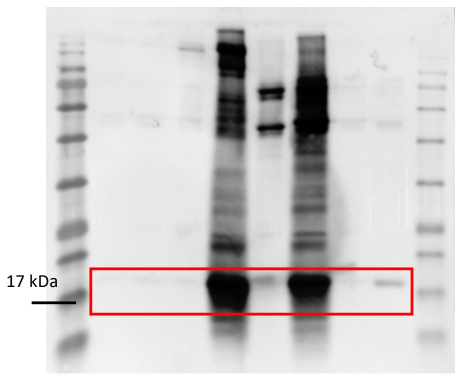

IB: phospho-tyrosine

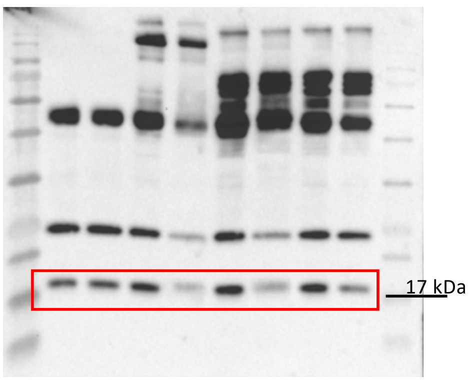

IB: MBP

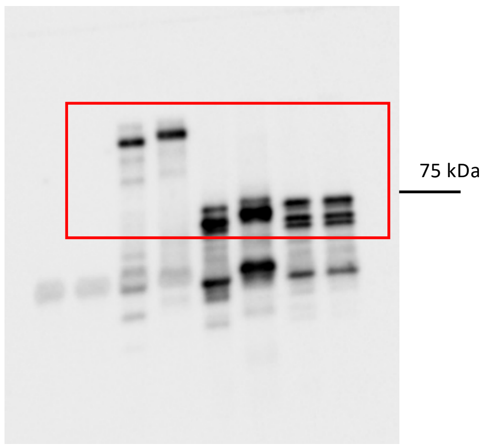

IB: V5

Figure 3B

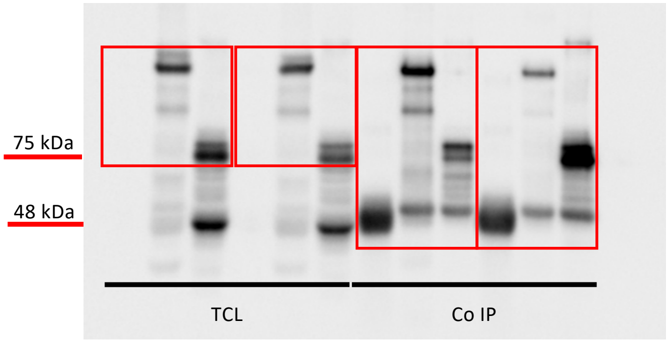

IB: V5

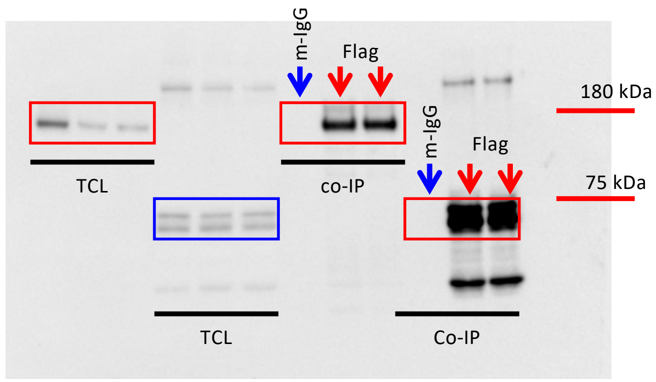

IB: Flag

Figure 3C

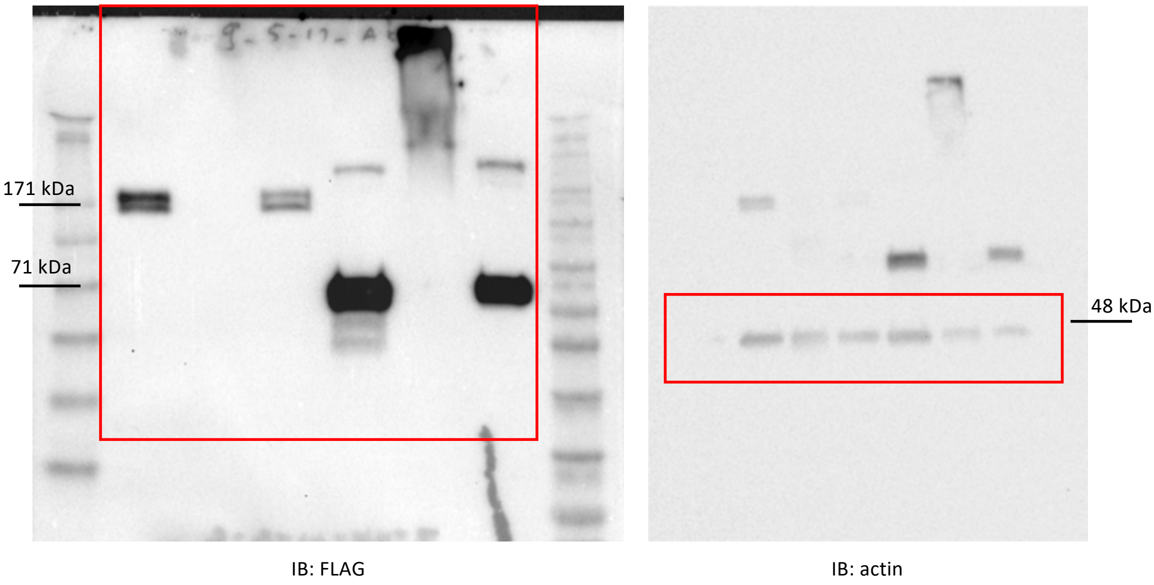

Figure 4C

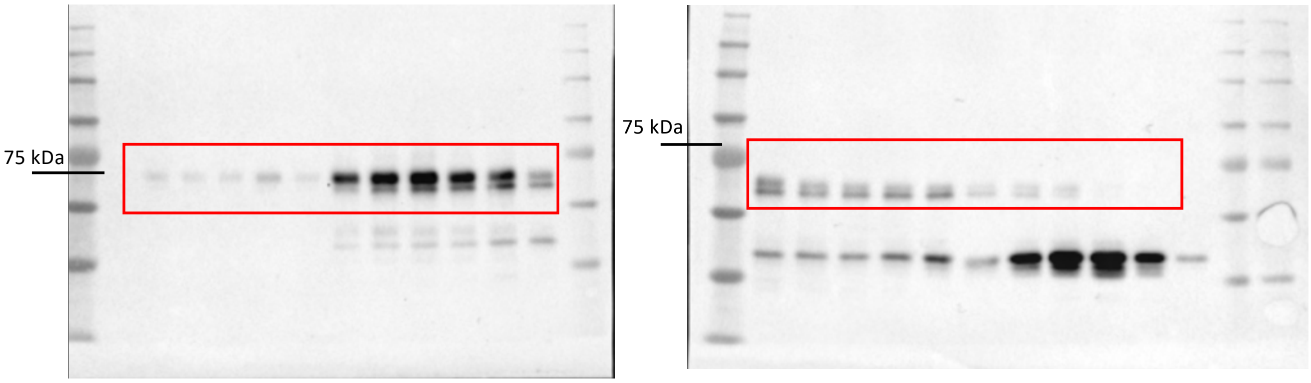

IB: V5
Samples: TFG-RET

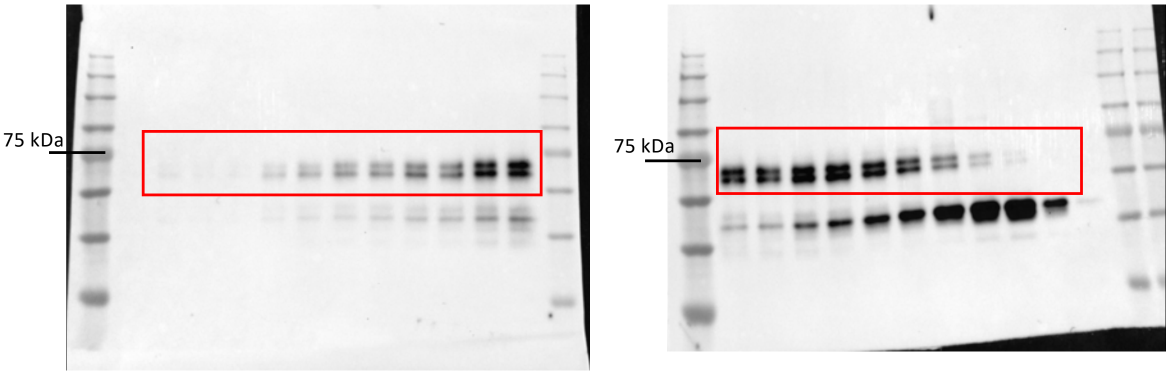

IB: V5
Samples: TFG-RET K14ER22ER23E

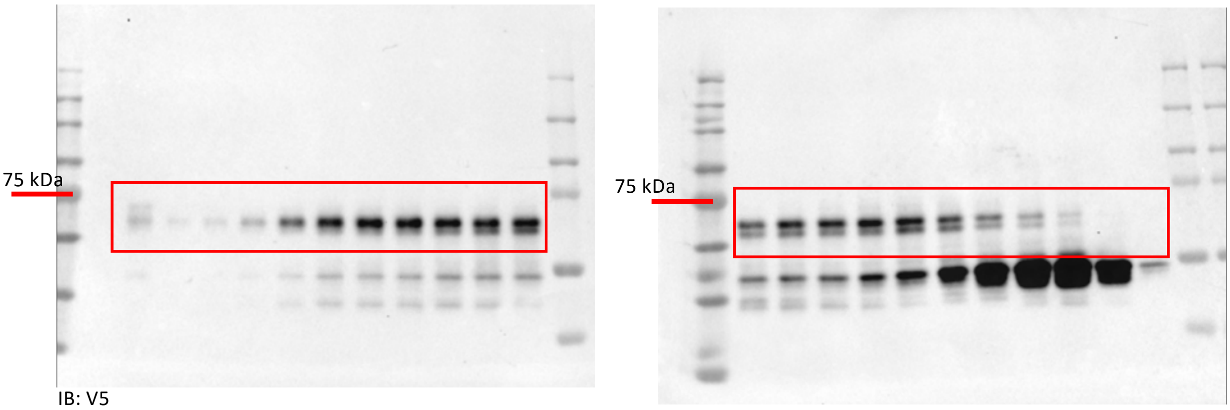

IB: V5
Samples: TFG-RET Δ97-124

Figure 4E

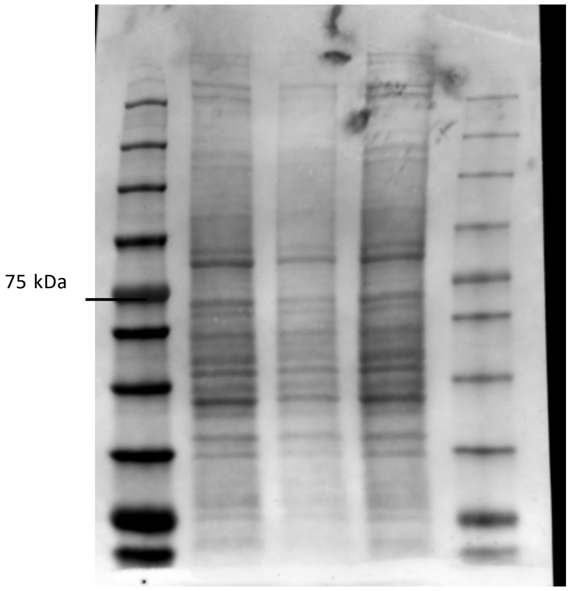

Ponceau S staining

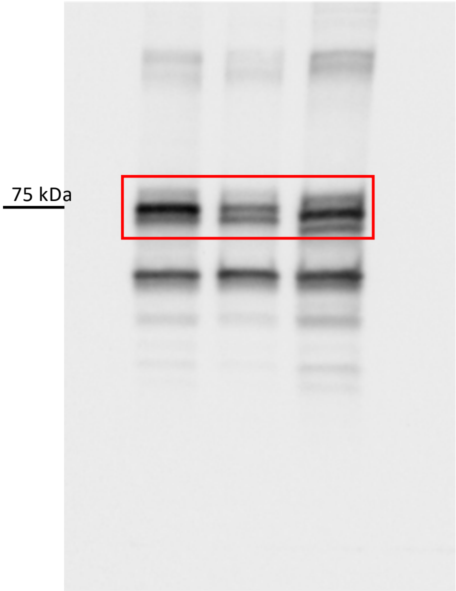

IB: V5

Figure 4F

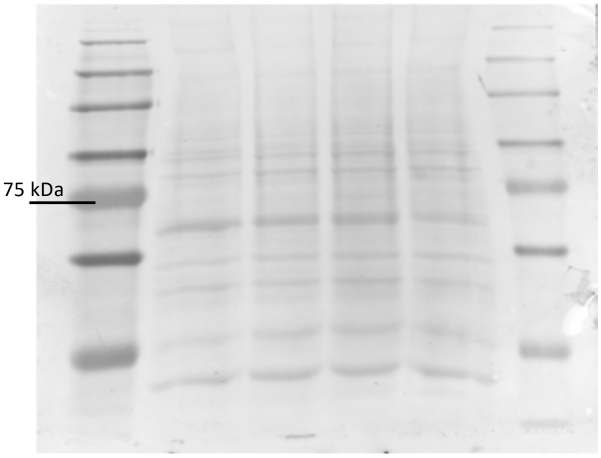

Ponceau S staining

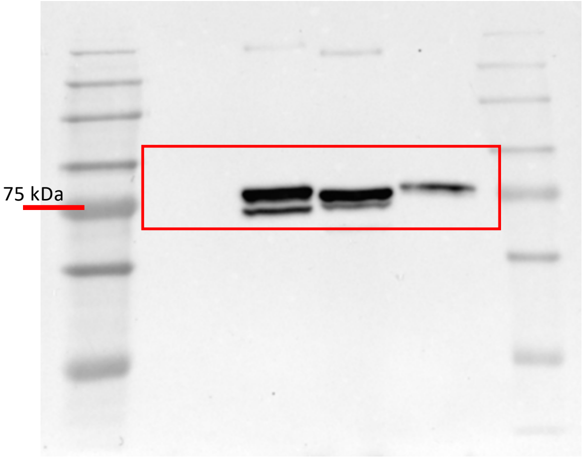

IB: Flag

Figure 5C

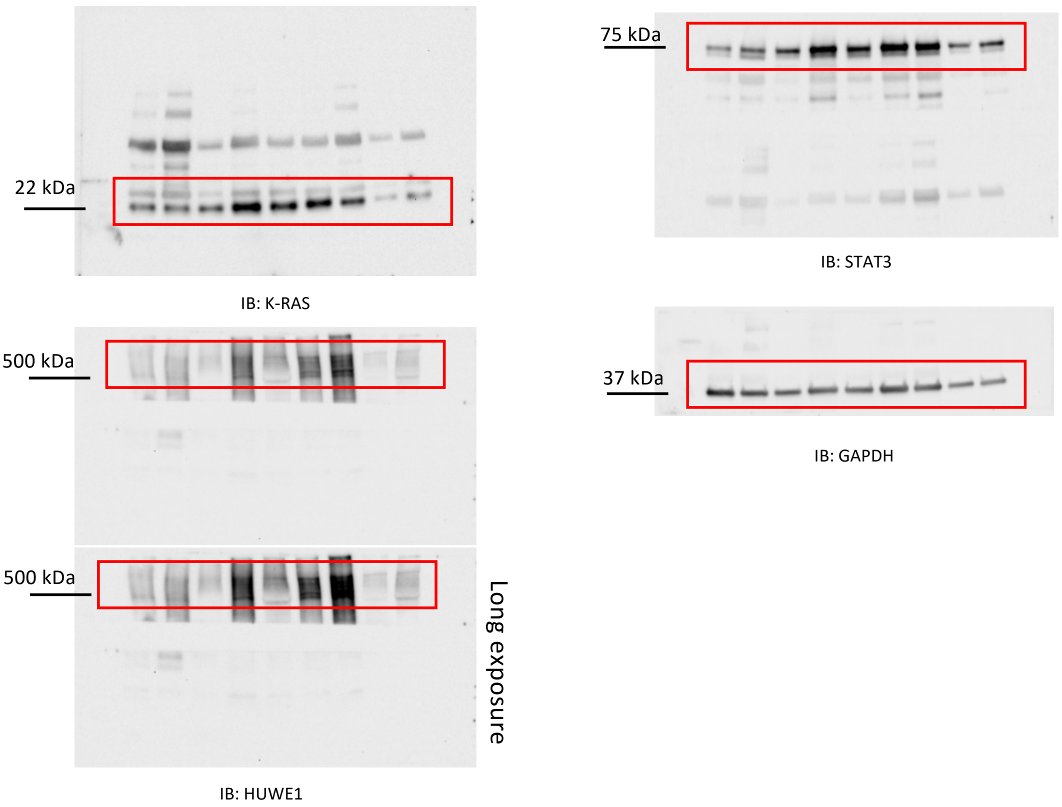

Figure 5D

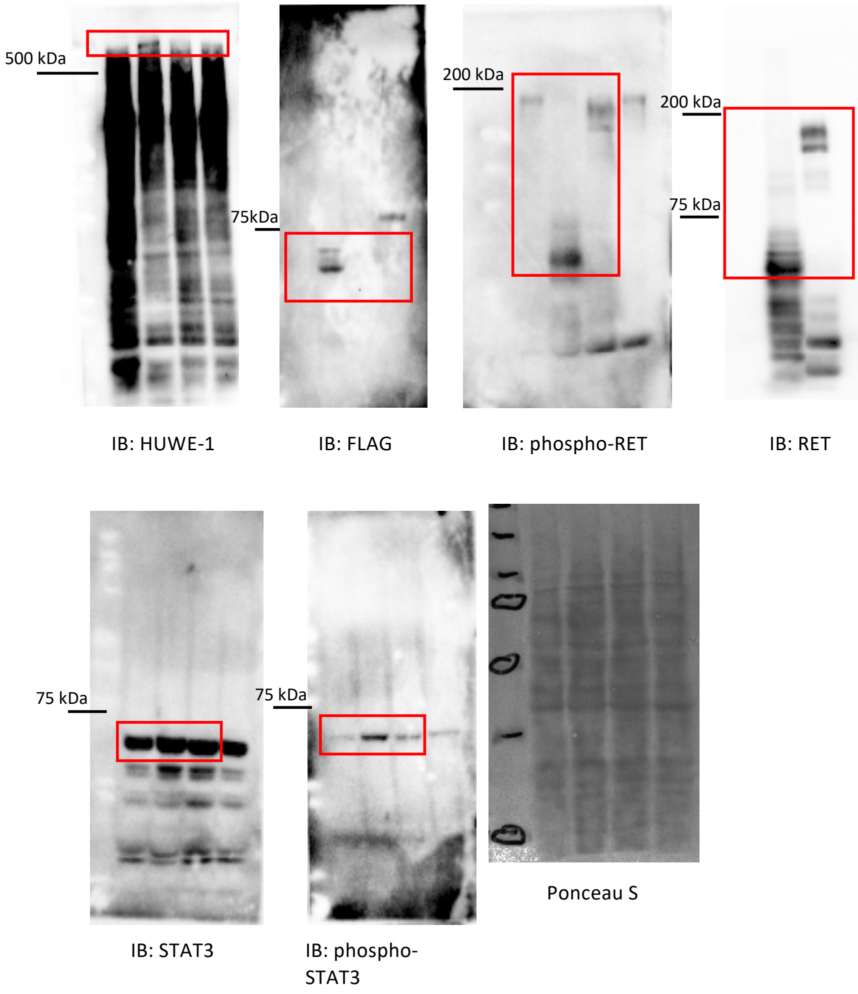

Supplementary Figure 3B

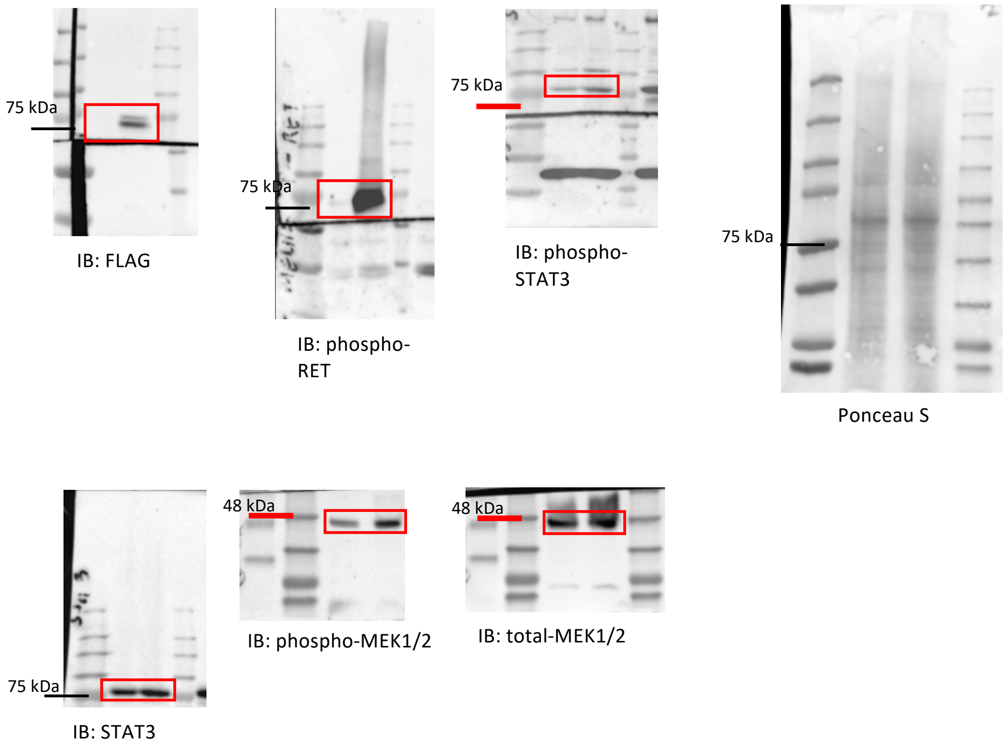

Supplementary Figure 3C

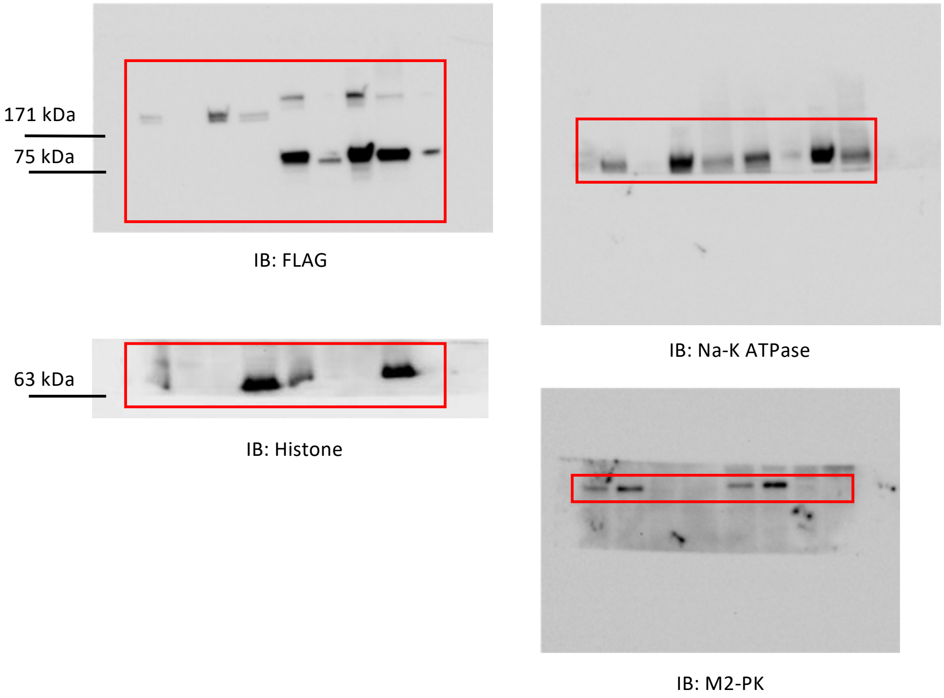

Supplementary Figure 4A

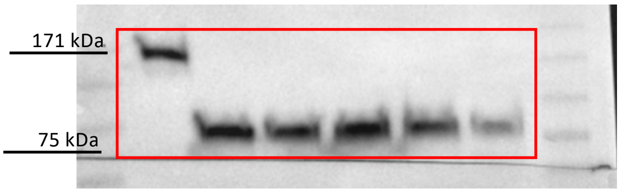

IB: phospho-RET

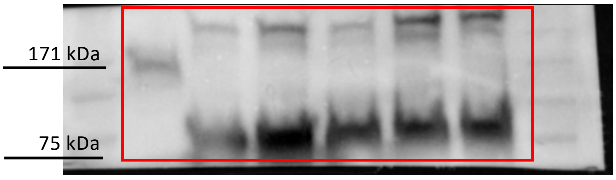

IB: V5

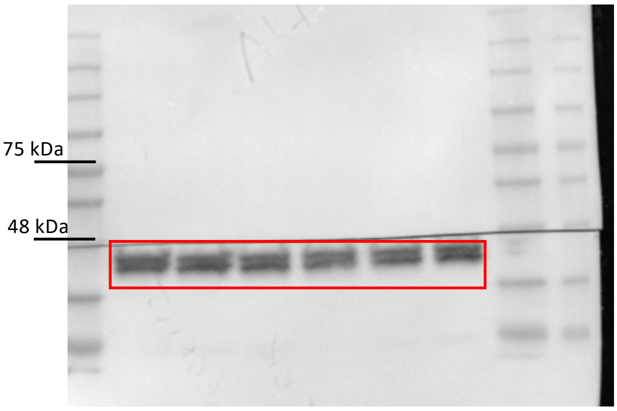

IB: ERK1/2

Supplementary Figure 4B

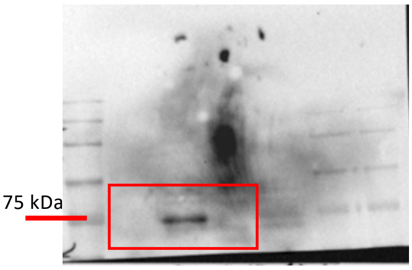

IB: phospho-RET

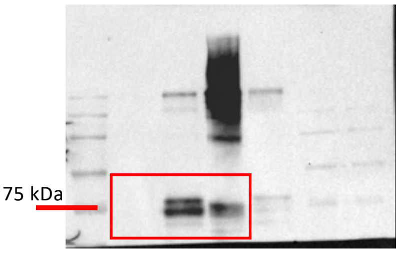

IB: V5

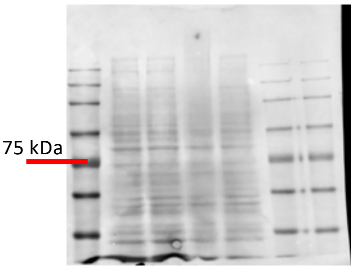

Ponceau S

Supplmentary Figure 4C

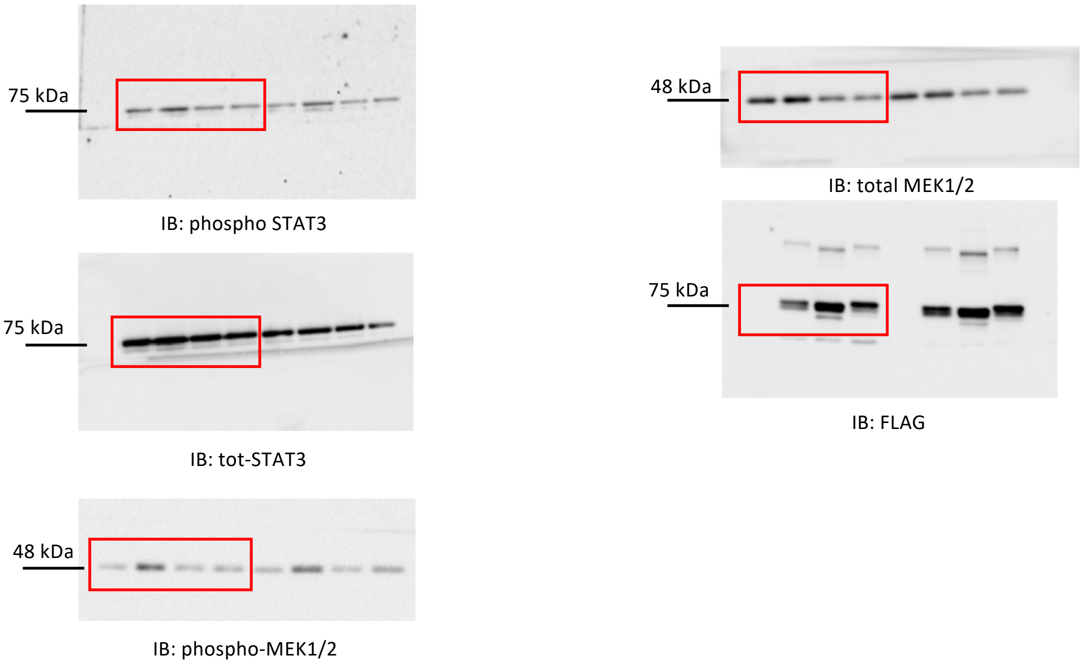

Supplementary Figure 4D

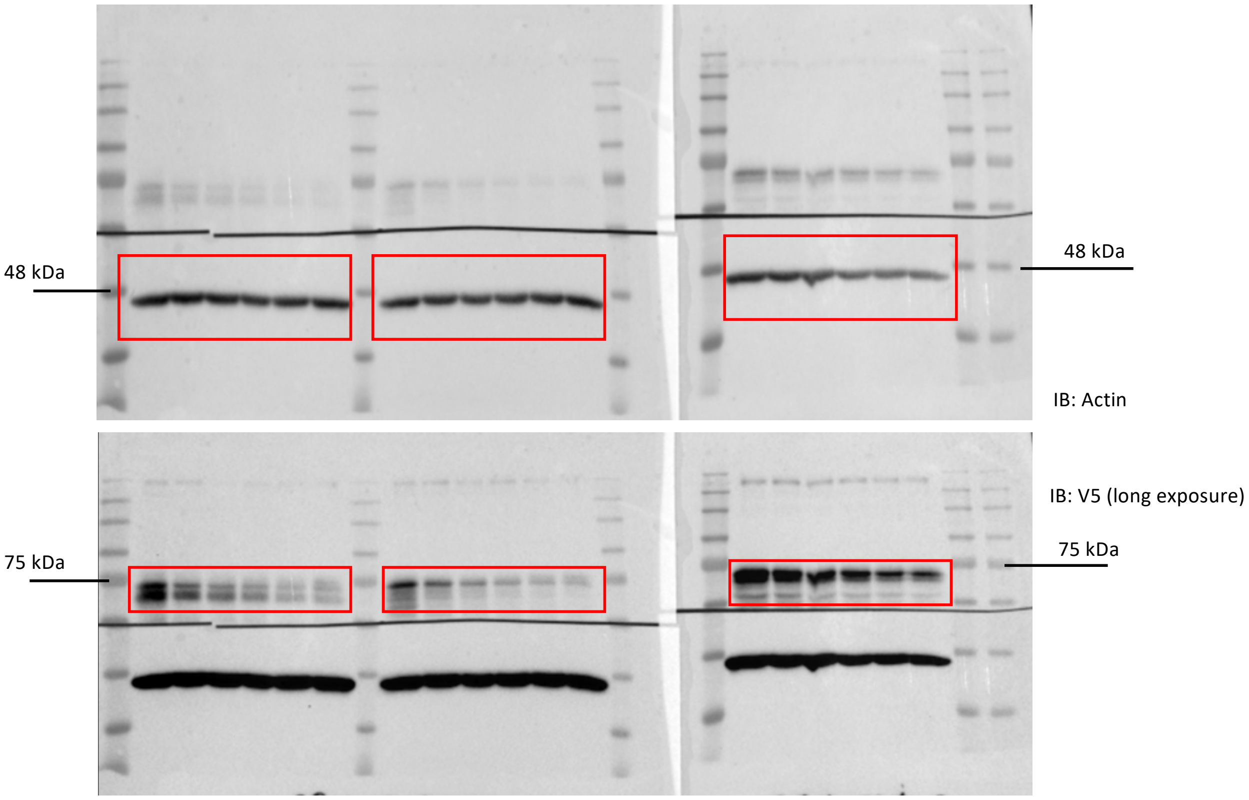

Supplementary Figure 5D

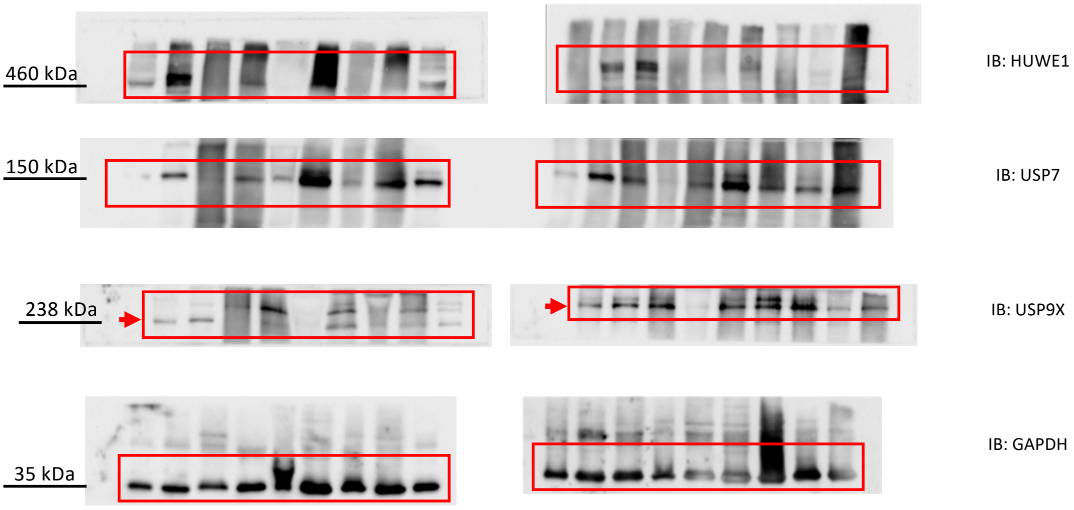

Supplementary Figure 5F

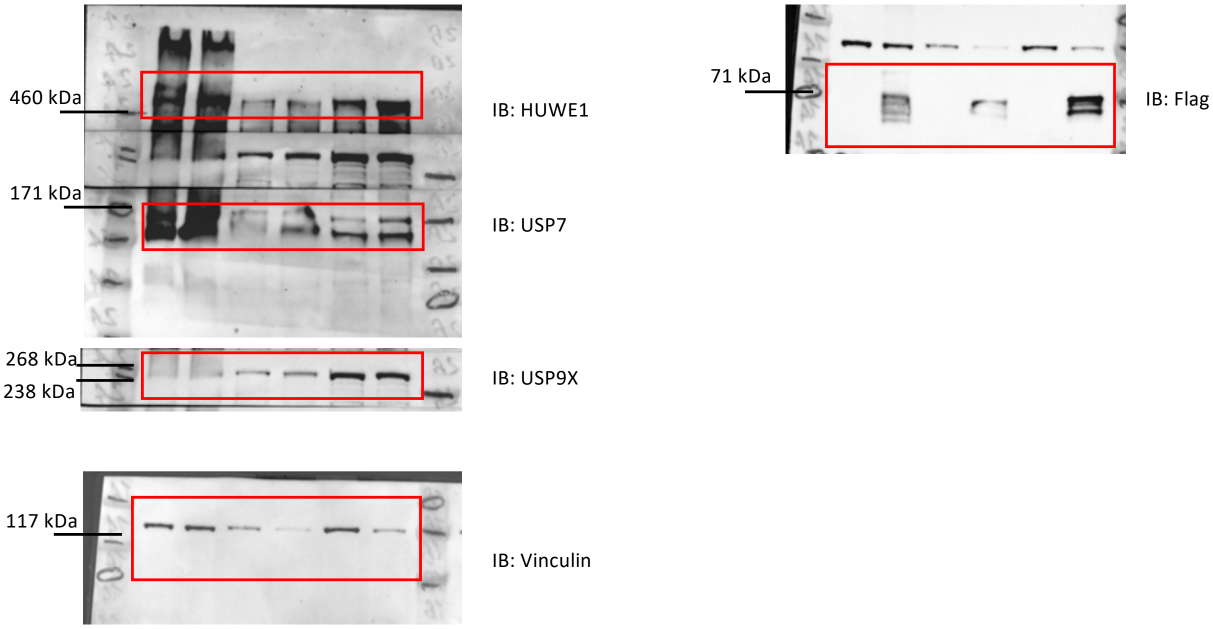

Supplementary Figure 6A

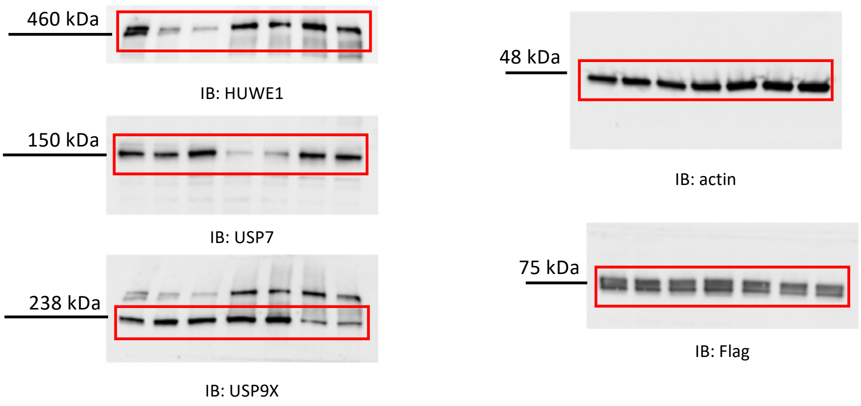

Supplmentary Figure 6D

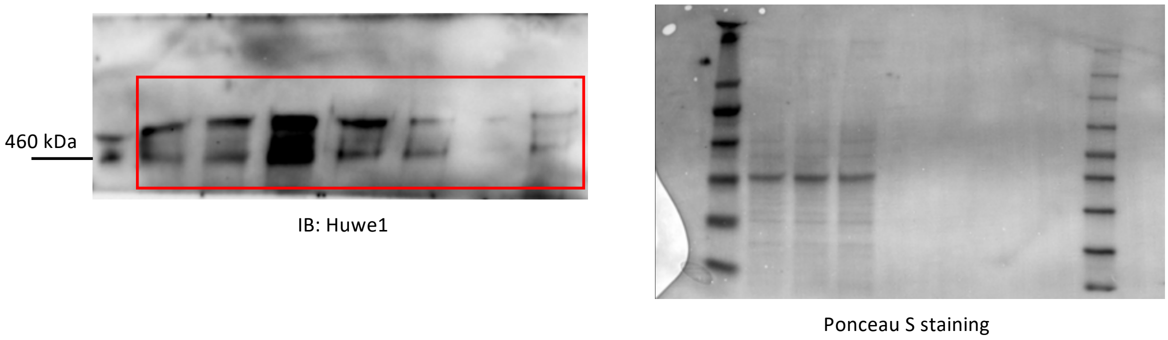

Supplement: Supplementary file 11 — Source Data [file 41467_2020_15955_MOESM11_ESM.pdf]
